# Internet-based cognitive behavioral therapy in children and adolescents with obsessive compulsive disorder: a feasibility study

**DOI:** 10.1007/s00702-021-02409-w

**Published:** 2021-08-25

**Authors:** Karsten Hollmann, Katharina Allgaier, Carolin S. Hohnecker, Heinrich Lautenbacher, Verena Bizu, Matthias Nickola, Gunilla Wewetzer, Christoph Wewetzer, Tord Ivarsson, Norbert Skokauskas, Lidewij H. Wolters, Gudmundur Skarphedinsson, Bernhard Weidle, Else de Haan, Nor Christan Torp, Scott N. Compton, Rosa Calvo, Sara Lera-Miguel, Anna Haigis, Tobias J. Renner, Annette Conzelmann

**Affiliations:** 1 Department of Child and Adolescent Psychiatry, Psychosomatics and Psychotherapy, University Hospital Tübingen, Osianderstr. 5, 72076 Tübingen, Germany; 2 Section for Information Technology, University Hospital Tübingen, Tübingen, Germany; 3 Child and Adolescent Psychiatry and Psychotherapy, Clinics of the City of Cologne, Cologne, Germany; 4 Department of Psychology (Clinical Psychology II), PFH-Private University of Applied Sciences, Göttingen, Germany; 5 Freelance Software Developer, Reutlingen, Germany; 6 Institute of Neuroscience and Physiology, University of Gothenburg, Gothenburg, Sweden; 7 Regional Centre for Child and Youth Mental Health and Child Welfare Faculty of Medicine and Health Sciences, Trondheim, Norway; 8 Faculty of Psychology, University of Iceland, Reykjavik, Iceland; 9 Regional Centre for Child and Youth Mental Health and Child Welfare, Trondheim, Norway; 10 Academic Center for Child and Adolescent Psychiatry, Amsterdam, The Netherlands; 11 Centre for Child and Adolescent Mental Health, Eastern and Southern, Division of Mental Health and Addiction, Oslo, Norway; 12 Division of Mental Health Services, Akershus University Hospital, Lørenskog, Norway; 13 Duke Child and Family Study Center, Durham, USA; 14 Department of Child and Adolescent Psychiatry and Psychology, Hospital Clinic of Barcelona, Barcelona, Spain; 15 CIBERSAM, IDIBAPS, University of Barcelona, Barcelona, Spain

**Keywords:** Obsessive–compulsive disorder, Internet-based psychotherapy, App, Children, Cognitive behavioral therapy

## Abstract

Cognitive behavioral therapy (CBT) is the first choice of treatment of obsessive–compulsive disorder (OCD) in children and adolescents. However, there is often a lack of access to appropriate treatment close to the home of the patients. An internet-based CBT via videoconferencing could facilitate access to state-of-the-art treatment even in remote areas. The aim of this study was to investigate feasibility and acceptability of this telemedical approach. A total of nine children received 14 sessions of CBT. The first session took place face-to-face, the remaining 13 sessions via videoconference. OCD symptoms were recorded with a smartphone app and therapy materials were made accessible in a data cloud. We assessed diagnostic data before and after treatment and obtained measures to feasibility, treatment satisfaction and acceptability. Outcomes showed high acceptance and satisfaction on the part of patients with online treatment (89%) and that face-to-face therapy was not preferred over an internet-based approach (67%). The majority of patients and their parents classified the quality of treatment as high. They emphasized the usefulness of exposures with response prevention (E/RP) in triggering situations at home. The app itself was rated as easy to operate and useful. In addition to feasibility, a significant decrease in obsessive–compulsive symptoms was also achieved. Internet-based CBT for pediatric OCD is feasible and well received by the patients and their parents. Furthermore, obsessive–compulsive symptomatology decreased in all patients. The results of this study are encouraging and suggest the significance of further research regarding this technology-supported approach, with a specific focus on efficacy.

Trial registration number: Clinical trials AZ53-5400.1-004/44.

## Introduction

Obsessive–compulsive disorder (OCD) is a frequent psychiatric disease of children and adolescents with a prevalence of 0.5–3% (Heyman et al. 2003; Zohar et al. 1992; Canals et al. 2012). Without adequate treatment, there is a high risk of chronification and development of further mental health problems in adulthood (Wewetzer et al. 2001; Peterson et al. 2001), accompanied by reduced socioeconomic status and social integration (Thomsen 2000; Piacentini et al. 2003). For this reason, it is of crucial importance to offer effective therapeutic help as early as possible. Extensive research shows that cognitive behavioral therapy (CBT) is the first line treatment for OCD (NICE 2005; AACAP 2012). CBT for children and adolescents with OCD should be centrally based on exposures with response prevention (E/RP) leading to habituation towards OCD triggering situations and stimuli (Walitza et al. 2021; McGuire et al. 2015; Rosa-Alcázar et al. 2015). Another argument in favor of such an approach is provided by the inhibitory learning theory (Craske et al. 2014). This assumes that the original association between the response-provoking stimulus and the unconditioned stimulus is inhibited by a newly learned, non-anxiety association (Mohr and Schneider 2015). This process appears to be impaired in children and adolescents with OCD (Geller et al. 2017; McGuire et al. 2016), leading to reduced treatment response (Geller et al. 2019). The inhibitory learning model propagates the importance of varying stimuli and contexts in exposures (Craske et al. 2018). Providing E/RP to children and young people with OCD in their natural environments rather than in a clinic setting could meet this recommendation and enhance treatment outcomes (Selles et al. 2021).

Nonetheless, the reality of administered care shows that children often do not get this state-of-the-art therapeutic approach (Goodwin et al. 2002). One obstacle is the fact that access to treatment is particularly difficult for children and adolescents in rural areas (Himle et al. 2006). Pediatric patients with OCD often do not receive CBT as outpatient treatment and even if they are treated with CBT, exposures are too rarely part of the treatment (Krebs et al. 2015; AACAP 2012). However, cumulative research suggests that therapeutically accompanied and individually graded exposures with response prevention should be carried out especially in triggering situations e. g. the homes of the patients (Hohagen et al. 2014; Peris et al. 2015). The reasons why E/RP is hardly used during CBT vary from practicability in everyday therapeutic life, negative assumptions of the therapists, therapist distress and the feeling of not being competent to conduct exposures (Pittig et al. 2019; Moritz et al. 2019). In summary, there is a big difference between treatment recommendations and their actual implementation in health care. This emphasizes the need to find new strategies to apply state-of-the-art approaches in a way that is practical for both patients and therapists.

The use of modern technologies in health interventions can offer the possibility to bridge this gap (Wolters et al. 2017) and help young patients to get the therapy they need. There is a wide range of treatments that can be subsumed under the generic term of digital health interventions (Hollis et al. 2017). In the following, the main focus is on the area of internet-based therapies. The corresponding interventions can be classified according to certain aspects, such as the amount of participation of a therapist in the intervention on the one hand, and the synchronous versus asynchronous exchange between patient and therapist on the other (Klein et al. 2018). Due to the prompt contact between therapist and patient during a videoconference, this is classified as a synchronous delivery of therapy, and communication, such as an exchange by text messages and e-mails as asynchronous. While usually no therapist is actively involved in self-management programs, there is extended contact with a practitioner during videoconferencing. An example of treatment without therapist contact is the self-management-program of Rees and colleagues (2016). This study also revealed one of the main limitations of such an approach: there was a very high dropout rate, which often occurs in self-management-programs (Richards and Richardson 2012; Herbst et al. 2012). Although the body of evidence has not conclusively been clarified yet, there are indications of an advantageous effect of human support on adherence and effectiveness (Domhardt et al. 2019). The most direct digital equivalent of face-to-face therapy could be an internet-based therapy including videoconferencing.

The number of studies on the internet-based treatment of pediatric OCD is still small, but there are already first indications that internet-based approaches are effective (Hollis et al. 2017; Ferreri et al. 2019): The study by Lenhard and colleagues (2017a, b) compared an internet-based CBT program with therapist support (email, telephone) and a waitlist control group for adolescents (ages 12–17 years, *n* = 67) and their families. There was a significant decrease in OCD symptoms in the treatment group after 12 weeks of treatment. The treatment was also superior to the waitlist (*d* = 0.69). Similarly, Storch and colleagues (2011) studied 31 children and adolescents (7–16 years) with OCD by randomly assigning patients to either family-based cognitive behavioral therapy via web-camera or to a waiting group. The therapy program consisted of 14 60–90-min sessions of CBT during a period of 12 weeks. Results showed that web-based CBT compared to the waitlist control was superior in all primary outcome measures, with a large between-group effect size (*d* = 1.36) at post-treatment. Comer and colleagues (2017) treated a total of 22 patients aged 4–8 years with a 14-week CBT program. The conditions “video-assisted therapy” and “face-to-face” treatment at the clinic were compared. Under both conditions there was a significant decrease of the OCD symptoms (video-assisted therapy *d* = 1.53 and face-to-face therapy *d* = 1.64), in the pre–postcomparison without any significant difference. In the 6-month follow-up, the treatment effects were stable.

These results are promising in terms of improving care for children and adolescents with OCD. However, subsequent studies are needed to confirm these initial effects, even more so given the wide heterogeneity of digital approaches. Considering the various aspects described, it can be assumed that internet-based cognitive behavioral therapy (iCBT) is particularly beneficial for the clinical picture of OCD, especially because it can ensure access to specialist treatment independent from where the children live. Telemedical approaches can shorten geographical distances and lessen issues of stigma and undersupply of specialists (Comer et al. 2017). In addition, iCBT provides an opportunity for therapists to more easily guide their patients in exposure exercises in the home environment and to examine, where avoidance behavior can be observed in everyday life. Through this telemedical approach in a realistic setting, psychotherapeutic treatment in patients' homes can improve the ecological validity of the therapy (Schiepek 2020; Patrão et al. 2020).

The primary aim of this study was to investigate the feasibility and acceptance of an internet-based intervention among children with OCD, their parents and the therapist. To implement this, various technological elements and devices, such as video conferencing, smartphone apps, Empatica wristband and data cloud, were linked in combination with cognitive behavioral therapy. Until the time of our study, this combined approach had not been used in the treatment of children and adolescents with OCD. Based on the German therapy manual of Wewetzer and Wewetzer (2012), we designed an iCBT-treatment guide for 14 therapy sessions, 13 of which are video-based. To our knowledge, there are no video-based therapy studies for this group of patients that have been conducted using a manual adapted exclusively for online therapy. Beyond the previous studies, the therapy materials are made available to the families in an online data cloud system. An additional extension is also the especially developed app for recording the extent of obsessive–compulsive symptoms, daily problems and mood through self- and parent-report. One advantage of this is that the experience of the symptoms is recorded directly in the natural environment, potentially improving the assessment’s ecological validity (Tilley and Rees 2014). Getting information about the OCD symptoms from several persons (multiple-informant assessment) offers the possibility to get a comprehensive clinical impression of the OCD and to adapt the therapy according to the needs of the patients. Since the assessments of patients and parents often differ (Storch et al. 2006), important information may otherwise be missing.

Another goal was to determine to what extent technical support by the study team is necessary and to assess the usability of the individual technical components by all users. We anticipated that the technical approach of our study will be feasible due to the ease of use. We expected a high acceptance as well as satisfaction with the internet-based treatment approach and that the OCD symptoms would improve through the therapy.

## Methods

### Participants

Nine patients and at least one of their parents took part in the study. Inclusion criteria were (1) a primary diagnosis of OCD as defined by the DSM-5 (APA 2013); (2) age between 7 and 17 years; (3) a total score of at least 16 on the Children’s Yale-Brown Obsessive–Compulsive Scale (CY-BOCS; Scahill et al. 1997); (4) ability to read and write in German for the participant and one parent; (5) family home equipped with an internet broadband connection, computer, HD webcam and speaker; (6) being medication-free or on a stable dose for a period of 6 weeks or more; and (7) indication for CBT. Exclusion criteria were the following: (a) IQ below 70; (b) severity of OCD symptoms, associated with a very low level of social functioning and/or aggressive behavior at home, making inpatient treatment necessary; (c) psychiatric comorbidity which is more impairing than OCD and makes participation clinically inappropriate (e.g. severe eating disorder, psychosis, autism spectrum disorder, ongoing substance abuse); (d) suicidal intent; and (e) ongoing psychotherapy.

We recruited outpatients at two study centers in Germany (Department of Child and Adolescent Psychiatry Psychosomatics and Psychotherapy in Tübingen and the Clinic for Child and Adolescent Psychiatry of the Cologne Hospitals). We also provided information on our website about the possibility of taking part in this study. In addition, attention was drawn to the study on the website of the German Society for Compulsive Diseases (DGZ, www.zwaenge.de). Furthermore, child and youth psychiatrists, pediatricians, and educational counselling centers in the states of North Rhine-Westphalia, Hessen, Rhineland-Palatinate and Baden-Württemberg were informed about the study by e-mail or postal letter. Patients were able to participate regardless of their place of residence in Germany. The recruitment took place from January 2017 until August 2018. The original planning comprised a sample size of 20 subjects. This was not achieved for various reasons. A central factor was that the proportion of potential candidates in the inquiries was well below 50%. Other reasons for exclusion were that a mental illness other than OCD was prevalent or that the obsessive–compulsive symptomatology was not present to a clinically relevant degree, that there was an indication for full inpatient treatment, or the children's and adolescents' motivation for therapy was insufficient. We then extended the recruitment phase, but had to close the study before reaching the planned number of subjects due to expiring funding. The project was approved by the regional ethics committee in Tübingen and Cologne and registered at clinicaltrials.gov (number: F.1331477).

**Fig. 1:**
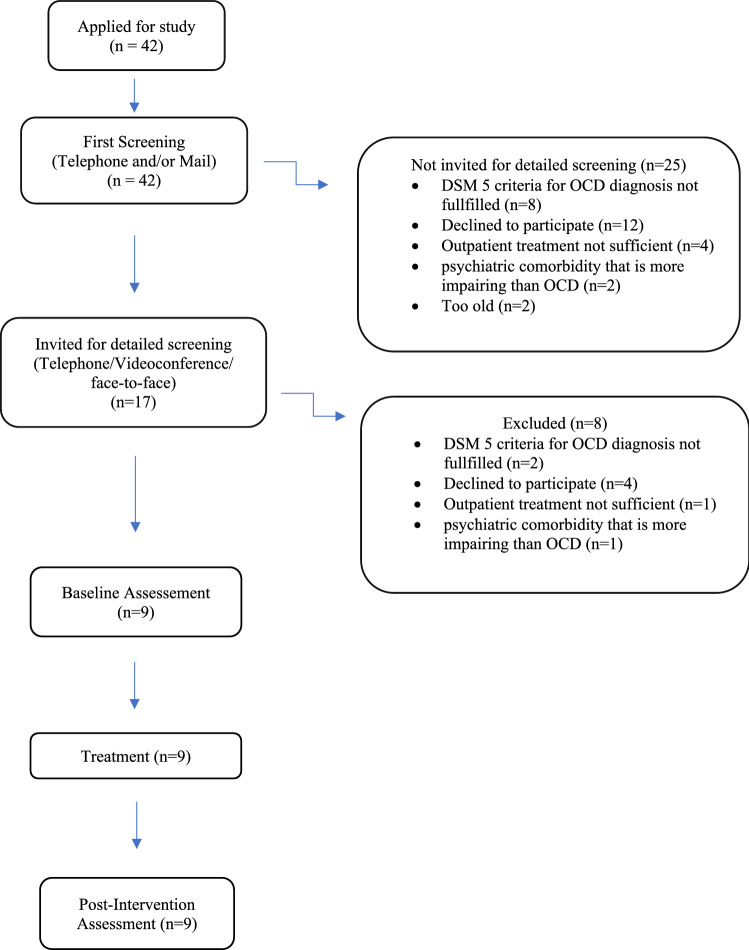
Study flow; *iCBT* internet-delivered cognitive-behavioral therapy

Figure 1 presents an overview of the study flow. The recruitment of the participants took place in three steps. Interested families were invited to contact study investigators by e-mail or telephone. A short telephone call was conducted, to explain the conditions of the study and to clarify the question of general eligibility. Given these, an appointment for a more in-depth interview was made, which took place by telephone, videoconference or face-to-face on site. If the inclusion criteria were basically met, the baseline assessment was conducted at a face-to-face appointment. Written informed consent was obtained from all participants and parents beforehand.

Table 1 gives an overview regarding the study population. The participants were on average 14 years. Six of the participants had already received psychotherapeutic treatment before, but only 3 of them because of OCD. When the study was conducted, 2 adolescents received a psychotropic medication. Two thirds of the participants had at least one additional comorbid psychiatric diagnosis besides OCD. Two of the participants lived within 20 km of one of the two study centers, another 2 within 50 km and another 2 within 70 km. The remaining 3 lived at least 200 km away. Table 1 gives further information on the demographic and clinical characteristics of the sample.

**Table 1:** Demographic and clinical characteristics of the study sample (*N* = 9)

Variable		*N*	%
Age (years)	*M* (*SD*)	14.11 (3.29)	
	Min–max	7–17	
Gender	Girls	2	22.2%
	Boys	7	77.8%
IQ	*M* (*SD*)	97.11 (7.57)	
	Min–max	83–104	
Distance between patient’s residence and treatment center (km)	*M* (*SD*)	127.11 (143.32)	
	min–max	12–425	
Current psychotropic medication	None	7	77.8%
	Fluvoxamine 150 mg	1	11.1%
	Sertraline 150 mg	1	11.1%
Earlier psychological treatments	None	3	33.3%
	CBT	2	22.2%
	PDT	1	11.1%
	Unspecified	3	33.3%
Reason of earlier psychological treatments	OCD	3	50%
	Unspecified	3	50%
Frequency of comorbid diagnoses (ICD-10)	Mild depressive episode (F32.0)	3	37.5%
	Specific reading disorder (F81.0)	1	12.5%
	Oppositional defiant disorder (F91.3)	1	12.5%
	Other childhood emotional disorder (F93.8)	1	12.5%
	Nonorganic enuresis (F98.0)	1	12.5%
	Stuttering (F98.5)	1	12.5%
Number of participants with 0–2 comorbid diagnoses	None	3	33.3%
	One	4	44.5%
	Two	2	22.2%
Duration of OCD symptoms (months)	< 18	3	33.3%
	> 18	6	66.7%

*CBT* cognitive behavior therapy; *PDT* psychodynamic therapy

### Measures

#### Measurements of feasibility, satisfaction and implementation

The *Summary Therapist Feedback Form* (STFF) assessed at posttreatment following a seven-point Likert scale (1 = “Not at all”, 4 = “Somewhat”, and 7 = “Very Much”) the therapists’ feedback about user-friendliness of the therapy materials, comprehensibility, and practicability of the treatment manual and whether all essential treatment elements were included in it (Crawley et al. 2013). In STFF, the therapist answered questions in regard to feasibility, after conclusion of therapy.

Following each therapy session, checklists referring to the session and the technology were filled in by the patients and their parents. Each question was rated on a Likert scale from 1 (not at all) to 9 (very much). In this checklist after the therapy session, the following questions were answered: “How helpful was the session for you?”, “How understandable were the session contents?”, “How confident are you that the therapy will help you/your child?”, “How convinced are you about the internet-based therapy?”. The checklist for the technology included the following questions: “How well did you cope with the app/cloud?” and “How helpful is the app?”.

The *Client Satisfaction Questionnaire-8* (CSQ-8) at posttreatment assessed the participant’s perceptions of the value of the services received (Crawley et al. 2013; Larsen et al. 1979).

*Questionnaire for the evaluation of the treatment* (FBB) is a German questionnaire that recorded how satisfied the participants were with the treatment. We reduced the total number of questions from 20 to 17, as 3 items covered circumstances that did not occur in the context of internet-based therapy (e.g., "I felt uncomfortable at the clinic"; Mattejat and Remschmidt 1998).

We developed a further *final therapy evaluation* based on relevant research to treatment evaluations (Carroll et al. 2007; Durlak and DuPre 2008) for the child, the parents, and the therapist. Each item was to be rated on a four-point Likert scale: I agree, I somewhat agree, I somewhat disagree and I disagree. It covered questions regarding the satisfaction with the therapy and aspects of implementation, such as adherence (intervention was delivered as intended—answered only by therapist), quality (how well different program components have been conducted), and program differentiation (unique features of the program).

#### Diagnostic instruments to record symptom change

The following instruments were assessed before (t0) and after the treatment (t1), except for the measure of intellectual ability, which was only conducted at the beginning of the study. The following diagnostic procedures were applied:

We assessed the *Children’s Yale-Brown Obsessive*–*Compulsive Scale* (CY-BOCS) as a semi-structured clinically administered interview and considered the gold standard in the assessment of symptom severity in paediatric OCD. The *Schedule for Affective Disorders and Schizophrenia for School-Age Children Present and Lifetime Version* (K-SADS-PL) as a semi-structured interview was used to diagnose childhood mental disorders (Kaufman et al. 1997). Depending on the age, the *Basic Intelligence Test Scale 1-Revised (CFT 1-R)* or *Scale 2-Revised* (CFT 20-R) was performed and served as a speech-free detection of fluid intelligence according to Cattell (Weiß and Osterland 2012; Weiß 2008). We obtained *Clinical Global Impressions-Severity (CGI-S)* as a widely used clinician rating of the global severity of psychopathology (Busner and Targum 2007). We used the *Clinical Global Impressions-Improvement (CGI-I)* as a clinician’s rating of the overall improvement in clinical presentation relative to the baseline assessment. The *Children’s Global Assessment Scale* (CGAS) was used as a clinician’s rating of the patient’s overall level of functional strain (Shaffer et al. 1983). Furthermore, we assessed the *Child Obsessive*–*Compulsive Impact Scale* (COIS-RC) as a self-report and parent-report questionnaire designed to assess the impact of OCD symptoms on the psychosocial functioning of children and adolescents in home, social, and academic environments (Piacentini et al. 2007). The *Child Behavior Checklist* (CBCL/4–18) as a parent-report measured a wide range of child behavioral and emotional problems (Achenbach 1994) together with the *Youth Self Report* (YSR/11–18) as self-report for children and adolescents (Achenbach 1994). DSM-5 anxiety symptoms were measured with the *Screen for Child Anxiety Related Emotional Disorders* (SCARED; Birmaher et al. 1997). Depressive symptoms were rated by the *Depression Inventory for Children and Adolescents* (DIKJ; Stiensmeier-Pelster et al. 2014). With the *Questionnaire for Measuring Health-Related Quality of Life in Children and Adolescents* (KINDL), we recorded the quality of life by a self-report of children and parents (Bullinger et al. 2008).

#### Measurements of physiological data

To record the children's physiological data, such as heart rate, electrodermal activity and sleep quality during therapy, we developed a smartphone app that was linked to a special wristband (Empatica). This wristband was worn by the children during several days and nights and exposure sessions.

### Procedures

The study described here was a single-armed, 14-session feasibility design. After telephone interviews ensuring eligibility, participants signed written informed consent and were invited to take part in the baseline diagnostic assessment at the clinic (t0). The patient and at least one parent participated in this session. When the conditions of inclusion were met, an introduction to the use of the technological devices took place at the same day. The assessment was conducted by a licensed child and adolescent psychotherapist who had extensive experience in the treatment of pediatric OCD. The therapist also performed the therapy and was, therefore, not blinded. The first session took place at one of the study centers at Tübingen or Cologne, the following 13 sessions were conducted online via videoconference with 90 min per session. After the treatment, the post intervention assessment (t1) followed in a time frame of at the latest 3 weeks after the conclusion of treatment.

### Intervention

Based on the CBT-manual by Wewetzer and Wewetzer (2012) and discussion within the expert network of international researcher on CBT in OCD, a treatment guide was developed. The implemented therapy materials contained information and work sheets, tailored to a child and adolescent target group as well as the parents. The exercise materials (adapted from the Wewetzer and Wewetzer manual, 2012) were supplemented by case studies with a psychoeducative character, as well as several separate work sheets, e.g., regarding the topic of relapse prevention.

Similar to traditional CBT treatment for OCD, iCBT took place in four stages. Stage I contained the development of a therapeutic relationship as well as psychoeducation regarding compulsions. In addition, together with the patients and parents, explanatory cognitive models were discussed and individually fitted to the patient (session 1). One of the goals of stage II was externalization, in which the patients practiced creating a distance to the contents of their obsessions. In addition, the foundation for cognitive intervention was established beginning with the discussion of strategies the patients had used so far to deal with the obsessive thoughts and how helpful these were. The aim was to motivate the patients to adopt new, functional approaches. Another focus was to explain the goals and procedures of E/RP, as well as creating a first hierarchy of triggering situations. In addition, a test-exposure took place (session 3). The central elements of stage III were the conduction of exposures (part of every session from session 4 on), as well as cognitive interventions (sessions 4–13). Participants were required to perform exposures at home, adolescents alone and children partially with the help of their parents. Stage IV focused on relapse prevention and concluded with a discussion whether further on-site treatment was necessary. Exposures continued, but the focus was now on conducting exercises in unpracticed situations without therapeutic assistance (sessions 13 and 14). Throughout the course of treatment, although not in every session, caregivers were included. Part of the family-centered interventions was consulting the parents in how to encourage healthy behavior in their child, and how to ensure that they are no longer included in the compulsions of their child. The patient’s homework consisted of working through work sheets, as well as conducting exposures and testing cognitive interventions throughout the daily routine. The practitioner was a psychotherapist with many years of experience in treating OCD in children and adolescents. The entire therapy was accompanied by a specially developed smartphone app, in which the children as well as the parents rated daily symptoms, daily hassles and mood. They also provided information about levels of distress before, during, and after exposures at home or within the therapy sessions. Moreover, the children and the families rated the weekly OCD-symptoms in the app.

### Technology and data security

As a prerequisite for participation, the families had to have internet access and a computer with a webcam and speakers at home, allowing a well-functioning conduction of videoconferences. The teleconferencing system *vidyo*^*®*^ was used for the therapy sessions. Materials were provided for the participants during the therapy session and made available via the secured online-cloud (BWSync&Share), provided by the federal state of Baden-Württemberg. Both patients and parents had their own password protected folder on the data cloud. Each family received a smartphone, which was configured to allow only the use of the study apps already installed. The questionnaire data of the app were regularly supervised by the therapist and evaluated to be used within the therapy sessions to adapt the therapy procedures. The physiological data collected by the app were not involved during the therapy and are, therefore, also not part of this manuscript. The data from the apps were safely encrypted and transferred into the Integrated Mobile Health Research Platform (IMeRa) of the University Hospital Tübingen, which is located in the secured clinic's network. The therapist could view the data in a browser-based application and used the information in the therapy sessions. In coordination with the IT security and data security experts of the study centers, all study routines were generated to verify data security. This procedure ensured that no data was permanently saved on the smartphone. The data were strictly pseudonymized so that re-assigning the data to the real names of the subjects by unauthorized third parties was not possible.

### Statistical analyses

Analyses were performed with SPSS 26. The analyses of the measurements of feasibility, acceptance and implementation were conducted descriptively, the change in symptoms were calculated through *t* tests. Here, Cohen's *d* is used as an effect size measure with a small effect starting at 0.2, a medium effect starting at 0.5, and a strong effect starting at 0.8 (Cohen 1988; Maher et al. 2013).

According to the suggestions of Skarphedinsson and colleagues (2017), a 35% reduction in CY-BOCS score was defined as treatment response. Remission is defined as a reduction of 55% at posttreatment CY-BOCS total score, respectively, lower than or equal to 11.

## Results

### Treatment satisfaction and acceptance

The results of the self-report questionnaire (*CSQ-8*) revealed that online-based treatment was well accepted by patients and their families and are detailed in Table 2

**Table 2:** Rates of perceived benefit from treatment by patients (Client Satisfaction Questionnaire—CSQ-8)

Item	*M* (SD)
(1) How would you rate the quality of care you have received?	1.89 (0.78)
(2) Did you get the kind of help you wanted?	3.22 (0.67)
(3) To what extent has the program met your needs?	2.11 (0.78)
(4) If a friend needed similar help, would you recommend the program to him/her?	3.33 (0.70)
(5) How satisfied are you the amount of help you have received?	3.44 (0.53)
(6) Has the help you have received helped you to deal more effectively with your problems?	1.78 (0.67)
(7) In overall, general sense, how satisfied are you with the help you have received?	1.67 (0.50)
(8) If you were to seek help again, would you come back to our program?	3.00 (0.71)

Anchors for Likert scale by question were as follows: Question (1) 1 = Excellent, 2 = Good, 3 = Fair, 4 = Poor; Questions (2), (4), and (8) 1 = No, definitely not, 2 = No, not really, 3 = Yes, generally, 4 = Yes, definitely; Question (3) 1 = Almost all of my needs have been met, 2 = Most of my needs have been met, 3 = Only a few of my needs have been met, 4 = None of my needs have been met; Question (5) 1 = Quite dissatisfied, 2 = Indifferent or mildly dissatisfied, 3 = Mostly satisfied, 4 = Very satisfied; Question (6) 1 = Yes, it helped a great deal, 2 = Yes, it helped somewhat, 3 = No, it did not really help, 4 = No, it seemed to make things worse; Question (7) 1 = Very satisfied, 2 = Mostly satisfied, 3 = Indifferent or mildly dissatisfied, 4 = Quite dissatisfied

The evaluation of the *FFB* showed that 6 participants described their satisfaction with the treatment as average (PR 26–75), one as above average (PR 76–90) and 2 as well above average (PR 91–100).

Regarding satisfaction with treatment, both patients and families indicated that they experienced therapy as helpful and understandable. The results of the *weekly questionnaires* regarding the interaction between patients, parents and the therapist are shown in Table 3. In the free text field of the questionnaire some parents noted as limitation that the questions in the app are not sufficiently tailored to the individual OCD symptoms.

**Table 3:** Weekly therapy session feedback

Item	Parents *M* (SD)	Children *M* (SD)
(1) How satisfied are you with the therapist?	8.74 (0.48)	8.76 (0.51)
(2) Did the therapist respond well to your problems?	8.55 (0.72)	8.58 (0.59)
(3) I was able to cope with the app	7.72 (1.06)	8.06 (1.24)
(4) I was able to cope with the cloud	7.64 (1.36)	7.56 (1.69)
(5) I found the app helpful	6.79 (1.02)	7.59 (1.27)
(6) I found the treatment helpful	7.37 (0.13)	7.77 (1.08)
(7) How comprehensible was the session content?	8.00 (0.12)	8.49 (0.75)
(8) How convinced are you that therapy can help you/your child?	7.61 (0.12)	7.27 (1.55)
(9) How convinced are you about Internet therapy?	7.56 (0.14)	7.61 (1.29)

Items rated on a nine-point Likert scale, where 1 = “very low”, 9 = “very high”

After therapy, all parents reported good understanding of what to do to support their children against OCD. Similarly, at the end of therapy, all children reported having a good understanding of how to manage their compulsions and how exposure exercises work. The results of the *final therapy evaluation* can be found in detail in Table 4. Parents and patients named the following aspects as advantages of internet-based therapy: “no travel times and expenses”, “good for people who live in the countryside and do not have access to treatment within a reasonable distance”, “performance of practical exercises”, “performing exercises at home”, “treatment by OCD expert”.

**Table 4:** Final therapy evaluation

Item	Parents agreed in %	Children agreed in %
(1) I liked it, that the therapy was carried out via the internet	75	89
(2) I think a therapy without the internet, where I had face-to-face contact with the therapist, would have suited me better	33	33
(3) Of the different things we did in therapy, I found helpful: explanations of what a compulsion is, how it occurs, and what to do about it	100	100
(4) I have a good understanding of what I can do to support my child against OCD	100	–
(5) I have well understood how the exposure exercises work	–	100
(6) I have well understood how to deal with obsessions	–	100
(7) The OCD-symptoms are weaker than before the treatment	91	89
(8) Family life has improved since the treatment	100	90
(9) My child/I would have needed more therapy sessions to learn how to get rid of OCD	83	78
(10) I was able to trust the therapist	100	100
(11) The therapist was interested in me/us and my/our problems	100	100
(12) I found it useful that the worksheets were exchanged and edited via the cloud	91	89
(13) I found it useful to have the app for feedback	83	100
(14) I found it difficult to use the program for video calls on the computer	17	11
(15) We had to interrupt therapy or started later, because the videoconference program did not work	17	55

Items rated on a four-point Likert scale, where 1 = “I agree”, 2 = “I rather agree”, 3 = ”I rather disagree” and 4 = “I don´t agree.”. We have taken the answers 1 and 2 as agreement as shown in the table

The assessment of the feasibility of the therapy from the therapist's point of view is shown in Table 5.

**Table 5:** Summary Therapist Feedback Form (STFF)

Item	*M* (SD)
How easy was it to understand the content of the manual?	7.00 (0.00)
How easy was it to conduct the treatment as outlined by the manual?	3.78 (1.30)
How user-friendly were the treatment materials?	5.89 (0.33)
Did the manual allow for enough flexibility?	2.44 (0.53)
Did you feel the 14 sessions were sufficient to accomplish all of the treatment goals?	1.78 (1.99)
Where there any unnecessary elements included in the manual?	1.11 (0.33)
Where there any important elements missing from the manual?	3.44 (2.51)

Items rated on a seven-point Likert scale, where 1 = “Not at all”, 4 = “Somewhat”, and 7 = “Very much”

### Need for technical support

23 times it was necessary to support the families with technical difficulties. The problems arose primarily when using the videoconferencing program. On several occasions, it was not possible to establish a connection at the beginning of the therapy session at the first attempt. In addition, there were isolated cases, where patients or parents had forgotten login details to the cloud or the smartphone, which then had to be recreated.

### Diagnostic instruments to record symptom change

The within-group *t* tests showed a significant decrease in symptom severity measured with the CY-BOCS. Comparing scores between pre-treatment (*M* = 25.67, SD = 5.00) and post-treatment (*M* = 16.78, SD = 8.3), statistical significant reduction in obsessive–compulsive symptomatology was found, *t*(8) = 6.06, *p* < 0.001, *d* = 2.02, corresponding to a large effect. Overall, the CY-BOCS total score decreased on average by 34% (8.7 points). Remission was achieved in 2 of 9 patients, 4 of 9 patients were considered treatment responders. The clinician rated-symptom severity (CGI-S) also revealed a significant reduction from pre-treatment (*M* = 4.89, SD = 0.93) to post-treatment (*M* = 3.44, SD = 1.51), *t*(8) = 4.27, *p* = 0.003, with a large effect of *d* = 1.43. Regarding the general improvement in clinical presentation (CGI-I), a positive change of the symptomatology could be observed in all patients; 6 of 9 participants (66%) were rated “much improved” or “very much improved” (*M* = 2.11, SD = 0.78). The improvement in terms of the level of social functioning (CGAS) from pre-treatment (*M* = 47.22, SD = 5.07) to post-treatment (*M* = 59.67, SD = 11.87) was also significant, *t*(8) = 4.01, *p* = 0.004 which corresponds to a large effect of *d* = 1.34. For the remaining outcomes (COIS-RC, CBCL, YSR, DIKJ, KINDL, SCARED) no significant changes between pre- and post-assessment were observed. The individual values are listed in Table 6.

**Table 6:** Diagnostic measures’ means (M) and standard deviations (SD) at pre- and post-treatment

Measures	Pre-treatment	Post-treatment	Statistics	
	*M* (SD)	*M* (SD)	*t* test	*p*
CY-BOCS				
Total score	25.67 (5.00)	16.78** (8.30)	*t*(8) = 6.06	*p* < 0.001
Obsessions	12.33 (3.12)	8.22** (4.21)	*t*(8) = 5.21	*p* < 0.001
Compulsions	13.33 (2.12)	8.56** (4.39)	*t*(8) = 5.65	*p* < 0.001
CGI-S	4.89 (0.93)	3.44 **(1.51)	*t*(8) = 4.27	*p* = 0.003
CGAS	47.22 (5.07)	59.67** (11.87)	*t*(8) = 4.01	*p* = 0.004
COIS-RC				
Parent rating	34.22 (24.86)	26.44 (22.25)	*t*(8) = 2.07	*p* = 0.07
Child rating	15.00 (16.12)	18.38 (21.45)	*t*(7) = .36	*p* = 0.73
CBCL				
Total score/problems	42.67 (20.55)	39.67 (21.58)	*t*(8) = 0.87	*p* = 0.41
Internalizing	13.89 (8.72)	13.67 (11.08)	*t*(8) = 0.10	*p* = 0.92
Externalizing	10.22 (7.67)	12.00 (8.90)	*t*(8) = 1.26	*p* = 0.24
YSR^d^				
Total score/problems	36.75 (23.02)	31.00 (20.51)	*t*(7) = 0.98	*p* = 0.36
Internalizing	11.38 (9.05)	10.88 (5.67)	*t*(7) = 0.23	*p* = 0.82
Externalizing	8.63 (8.45)	8.13 (8.97)	*t*(7) = 0.31	*p* = 0.77
DIKJ	27.11 (4.28)	26.56 (7.55)	*t*(8) = 0.22	*p* = 0.83
KINDL				
Parent rating life quality	64.15 (14.04)	68.75 (14.82)	*t*(8) = 1.17	*p* = 0.28
Child rating	74.42 (12.45)	72.19 (8.67)	*t*(8) = 0.47	*p* = 0.65
Scared				
Parent rating	16.89 (10.66)	15.33 (8.56)	*t*(8) = 0.35	*p* = 0.74
Client rating	15.25 (9.74)	13.25 (8.94)	*t*(7) = 0.61	*p* = 0.56

*CY-BOCS* Children’s Yale-Brown Obsessive–Compulsive Scale; *CGI-S* Clinical Global Impressions-Severity; *CGAS* Children’s Global Assessment Scale; *COIS-RC* Child Obsessive–Compulsive Impact Scale; *CBCL/4–18* Child Behavior Checklist; *YSR/11–18* Youth Self Report; *DIKJ* Depression Inventory for Children and Adolescents; *KINDL* Questionnaire for Measuring Health-Related Quality of Life in Children and Adolescents; *SCARED* Screen for Child Anxiety Related Emotional Disorders

*p < 0.05, **p < 0.01, *p* values refer to comparison from pre- to post-treatment

## Discussion

The aim of this study was to investigate the feasibility and acceptance of a telemedical treatment approach for OCD in children and adolescents. The results of our study underline that both are given. Likewise, we have focused on the application of specific therapeutic materials and strategies for online-based treatment that have not been explicitly developed in this form before.

Demand for study participation was high. Approximately 40 inquiries for interested parties were received, with an average distance to study sites of 127 km. This number suggests that families searched, or had to search, well outside the immediate service radius of the health care system close to home to obtain a treatment slot.

The survey of patient and parent satisfaction with treatment revealed high levels of agreement among both patients and parents. In addition, almost all patients would recommend iCBT to a friend with similar problems. When looked at in a differentiated way, the data also show that the quality of treatment was rated as very high by the patients. Furthermore, it should be positively emphasized that patients and parents experienced the treatment as helpful and felt that the content was conveyed in a way that was easy to understand. Further evidence of satisfaction with iCBT is that there was no drop out and all families completed the treatment. Our findings are comparable to those from other technology-based treatments for pediatric OCD (Storch et al. 2011; Lenhard et al. 2017a, b; Comer et al. 2014, 2017). In a therapist-supported online therapy program (Lenhard et al. 2017a, b), more than 60% of patients rated treatment as “good” or “very good.” Satisfaction ratings were also high or even higher for videoconference-based approaches (Comer et al. 2014, 2017; Storch et al. 2011).

Related to the question of iCBT acceptance, as with satisfaction, the response was positive. Both patients and parents were convinced by the internet-based treatment, as was shown by the survey conducted after each therapy session. The values from the final therapy evaluation also highlight these results. The fact that the treatment took place via the Internet was rated positively by 89% of the patients, and by 75% of the parents. However, the question of whether face-to-face treatment would have been preferred to iCBT is particularly interesting. The majority of patients and parents answered this in the negative (67% each). Only Lenhard and colleagues (2017a, b) addressed this question as we did. However, their focus was not exclusively, on whether face-to-face treatment would have been preferred overall, but also on whether there should have been repeated face-to-face contact with a therapist during the course of treatment. Only a very small number of patients (4%) would have preferred face-to-face treatment, but at least half would have preferred occasional meetings with a therapist. Further research on this topic should follow to draw valid conclusions on the form and extent of contact desired by patients.

A significant decrease in obsessive–compulsive symptoms was found in addition to the feasibility and acceptance of a telemedical treatment. To date, few studies exist on this issue, and our positive results encourage further research in this area of digital health interventions for pediatric OCD. Two of the nine patients met the remission criteria after treatment, and a total of four could be classified as treatment responders. The fact that such effects were illustrated despite the small sample size underlines the potential efficacy of the telemedical approach. The results point in the same direction as those of existing Randomized Controlled Trials (RCT) in which treatment was technologically assisted (Turner et al. 2014; Storch et al. 2011; Lenhard et al. 2017a, b; Comer et al. 2017). Concurrent with the decrease in obsessive–compulsive symptomatology was an improvement in the psychosocial functioning levels of the children and adolescents. This is significant, because impairment in everyday life due to the compulsions has been documented (Piacentini et al. 2003; Valderhaug and Ivarsson 2005), but the amount of evidence for children and adolescents that treatment also has a positive effect on the level of functioning is still limited (Thomsen 1995; Turner et al. 2014; Storch et al. 2013, among others). Therefore, this study has targeted this issue.

In addition, it can be deduced from the information provided by the subjects that some of the patients would like to have a combination of digital elements and personal contact with the practitioner. This approach is referred to as blended treatment (Wentzel et al. 2016). In this case, the advantages from both treatment paths are used: Digital interventions can reduce the lack of specialized therapists and also facilitate access to helpful CBT treatment approaches. In this context, an important question for future studies would be the extent to which direct therapist support is needed to ensure optimal treatment success while maintaining cost-effectiveness of treatment. In online-only treatment without therapist support, high dropout rates and low treatment motivation were observed (Pearcy et al. 2016). In contrast, increased therapist contact, e.g., through scheduled phone calls, was associated with lower dropout rates, higher treatment motivation regarding self-performed exposures with response prevention, and greater improvement in obsessive–compulsive symptoms and thus higher effect sizes (Kenwright et al. 2005; Pearcy et al. 2016).

Among the aspects of treatment that families found particularly helpful were the therapist-guided exposure exercises in the domestic environment. These were also experienced as beneficial by the therapist and could be optimally implemented during the therapeutic sessions due to the telemedical treatment approach. Through this, the greatest symptom actualization was achieved. Despite certain limitations (among others, only a limited section of the exercise scene can be seen by the therapist due to the webcam), video conferencing provides the opportunity to accompany the patients in their living environment and to provide them with therapeutic support in precisely those situations that limit them most in everyday life. The inhibitory learning model propagates the importance of varying stimuli and contexts during exposures (Craske et al. 2018). Thus, even from this perspective, it makes sense to conduct exposures with patients in their natural environment somewhat than in a clinic setting. Guidelines for the treatment of OCD also recommend conducting exposures in the immediate place, where the compulsions occur (Walitza et al. 2021), and higher remission rates and satisfaction scores have been achieved (Selles et al. 2021).

With respect to seriously ill children and adolescents, the study illustrated that the implementation of the planned content in sessions 7–12 was not possible in full for all patients. A review of the therapy protocols revealed that particularly severely ill patients (CY-BOCS total score > 28) had difficulty engaging in exposures consistently. This resulted in a lower number of exercises for them; there was also evidence of avoidance behavior during the exercises. Combined, these factors are likely to have led to a poorer outcome, although it is not yet clear which of these factors play which role (Kircanski and Peris 2015).

None of the studies published to date for the child and adolescent sector comment on the questions of how well the technical aids (e.g., videoconferencing program) worked and how easy the participants found it to use them (usability). However, investigating this is necessary to draw conclusions about whether and, if so, how the technology used and its usability influenced treatment (Yogarajah et al. 2020). We conducted an evaluation to this end. This revealed that the various components of the technical equipment were positively evaluated with regard to the above-mentioned aspects. However, in accordance with our observations, specifically the patients at the final assessment indicated that there had been difficulties in using the videoconferencing program. In general, these occurred when starting the program during the first therapy sessions and required support from the therapist to resolve. Recording needs for technical support is useful for several reasons. From a health economic perspective, therapy services that are primarily delivered independently by patients are in principle less expensive to use than face-to-face therapies due to the reduced time commitment of psychotherapists (Lenhard et al. 2017a, b). However, if the technical support is provided by the psychotherapists themselves, the resulting time commitment must be considered in terms of costs. In addition, the needs assessment can be used to determine which qualifications should be present in a treatment team in addition to psychotherapeutic expertise.

Although the results are promising, our study is not without limitations. The original planned sample size of 20 subjects could not be reached in the study, because less than half of the potential candidates were suitable for the study or there was no OCD as main diagnosis. Despite an extension of the recruitment phase, the study had to be terminated before reaching the planned number of subjects due to expiring funding. Due to the lack of a control group, only limited conclusions can be drawn about the efficacy, and a statement about the stability of the effects is not possible, because no follow-up study was conducted. A blinding of the participants and especially of the diagnostician was not possible due to the single-arm study design. We noticed that the decrease in CY-BOCS scores as well as the rate of responders and patients in remission was lower than in the other technique-based studies on compulsions in childhood and adolescence. Given the background described above, this circumstance cannot be interpreted conclusively, but we nevertheless critically examined the data for possible clues to causation.

In summary, the results suggest that internet-based psychotherapy is well received by families and reduces symptoms in children with OCD. The COVID-19 pandemic showed us how significant internet-based interventions are, making this option a first-time implementation for many treatment providers. Guidance was quickly produced on what to look for generally in the treatment of OCD under COVID-19 (Fineberg et al. 2020), what opportunities digital therapy offers, and what aspects to consider when using it from a privacy and therapeutic perspective (Allgaier et al. 2020). Our study supports the usefulness of this approach with data; however, further studies with control groups should follow. To provide the appropriate evidence of efficacy, we designed and have already been able to start an RCT with a waiting group control design. In addition, it is certainly useful to test blinding designs and stepped-care approaches in trials (Aspvall 2020) and the extent of necessary direct interaction with the therapist. Overall, online-based approaches should also be pursued and investigated in OCD, but also in other disorders to provide early or timely access to experts for patients independent of location. Furthermore, research on efficacy factors and side effects of internet-based methods would be relevant.

## Data Availability

All data are available by the authors upon request.
